# Anthrax outbreaks: an upcoming concern – commentary

**DOI:** 10.1097/MS9.0000000000000282

**Published:** 2023-03-25

**Authors:** Kamran Zaman, Aroop Mohanty, Ranjit Sah

**Affiliations:** a Indian Council of Medical Research (ICMR)-National Institute of Traditional Medicine, Belagavi, Karnataka; b Department of Microbiology, All India Institute of Medical Sciences (AIIMS), Gorakhpur, India; c Dr. D.Y. Patil Medical College, Hospital and Research Centre, Dr. D.Y. Patil Vidyapeeth, Pune, Maharashtra, India; d Tribhuvan University Teaching Hospital, Institute of Medicine, Kathmandu, Nepal

Highlights

- Anthrax is a bacterial toxin-mediated zoonotic illness.
- It can be contracted by handling, consuming, inhaling, or injecting *Bacillus anthracis*-contaminated animal byproducts, such as skins, wool, or meat, or by-product-contaminated objects and fomites.
- *B. anthracis* can be used as a bioweapon.

*Dear Editor*,

Many Americans’ understandings of the anthrax threat are linked directly to the attacks of October 2001 through exposure to intentionally contaminated mail. Following this, *Bacillus anthracis* was listed as a tier 1 select agent by the U.S. Department of Health and Human Services in view of its risk of intentional misuse for mass casualties and causing serious threats to public health and safety. Following this incident, several incidences of anthrax cases have been reported worldwide by accidental or intentional release of spores^1^.

Recent reports of sporadic anthrax cases from Santo Niño (Cagayan, Philippines) and Maara (Tharaka-Nithi County, Kenya)^2^,^3^ have raised concerns over the preparedness against occupational or environmental exposures to *B. anthracis*. Similarly, new reports of its possible use in the Russia–Ukraine war have also threatened the health authority and policymakers with regard to its intentional use as a bioweapon^4^.

Anthrax is a bacterial toxin-mediated zoonotic illness that can be contracted by handling, consuming, inhaling, or injecting *B. anthracis*-contaminated animal byproducts, such as skins, wool, or meat, or by-product-contaminated objects and fomites. The agent has also undergone significant research as a bioweapon and a deliberate aerosol discharge of spores leading to public health threats^5^.

Within the genus *Bacillus*, *B. anthracis* is the only pathogen that is a permanent (obligate) pathogen and is Gram-positive and rod-shaped. The term ‘monomorphic species’ refers to *B. anthracis* isolates that are nearly genetically and phenotypically identical, regardless of the source or location. Only nonquantifiable or semiquantifiable traits, such as colony morphology, flocculation in broth culture, cell size, LD50 in animal experiments, and similar traits, can be used to phenotypically identify different strains^1^,^5^. Anthrax-causing agents are naturally present in soil and frequently infect domestic and wild animals worldwide. Ingesting spores from polluted soil, vegetation, or water might cause the animals to contract the disease. Most often, spores are produced when carcasses are opened by scavengers leading to air exposure of bacilli in blood and body fluids. Because they are extremely resilient, their spores contaminating the soil can persist for many years^5^.

Various animal species are more or less susceptible. Pigs and other carnivores have higher resistance and frequently acquire a subclinical illness, whereas cattle, sheep, and horses are particularly susceptible. The resistance of scavengers is often high. Animals normally take 3–7 days to fully incubate (range 1–14 days). A 20-day incubation period for World Organization for Animal Health (WOAH, founded as OIE) international trade regulations is required^5^.

Clinical anthrax symptoms in people typically correspond to the method of infection, whether it is cutaneous, inhalational, gastrointestinal, or injectable. Any anthrax transmission route may become complicated by meningitis, or it may develop without any obvious infection source^5^.

*Cutaneous anthrax*: The most prevalent type of anthrax typically develops after exposure to contaminated material or during the butchering of an animal that has died from anthrax. A breech in the skin increases the likelihood of developing this condition, which typically starts as a cluster of small blisters or lumps that itch, grow, and eventually turn into an ulcer with a black center.

*Inhalation anthrax*: This form of disease develops when bacillus spores are directly inhaled during bioterrorism incidents or when contaminated hides are processed in the leather industry. Clinical symptoms include a fever, chills, cough, chest pain, nausea or vomiting, headache, and weariness. Later, the person may also experience shortness of breath and confusion.

*Gastrointestinal anthrax*: This condition occurs when raw meat from an animal that has died of anthrax is consumed. Clinical symptoms include fever, chills, nausea or vomiting, abdominal discomfort, and diarrhea. A scratchy throat, trouble swallowing, and neck enlargement in a small number of instances may indicate a pharyngeal variant of the disease.

*Injection anthrax*: This illness kind is typically found in intravenous drug users who inject spore-contaminated illegal narcotics into their bodies. The person exhibits clinical characteristics resembling cutaneous anthrax but has a high risk for systemic dissemination.

*Management*: Blood, skin lesions, exudates, tissue, and bodily fluids are some of the specimens that can be used to diagnose anthrax in both humans and animals. Prior to starting antibiotic therapy, specimens must be collected^6^.

Antibiotics are effective in treating all anthrax infections. Based on the medical history and physical examination, physicians can choose whether oral or intravenous antibiotics are necessary. Another option for treatment is anthrax antitoxin, which targets anthrax toxins in the body but must be administered in conjunction with antibiotics. Serious instances of anthrax necessitate hospitalization and may call for extensive therapies such as mechanical breathing support, blood pressure support, and excess fluid drainage^5^,^6^.

In a systematic review by Hendricks *et al*.^6^, the survival rates for nonsystemically ill versus systemically ill adult people who got no treatment, one antimicrobial without antiserum, at least two antimicrobials without antiserum or antiserum or antitoxin alone were 48% versus 26%, 97% versus 73%, 100% versus 57%, and 96% versus 74%, respectively.

*Prevention*: The key measures for managing animal outbreaks are better case surveillance, prophylaxis, vaccination, and quarantine in outbreak settings, restriction of access to suspected sources (pastures or feed), proper carcass disposal, quick diagnosis and treatment of infected animals, appropriate disposal of the carcass, and through disinfection of contaminated materials and areas^5^.

Rapid identification of the outbreak animal source, humans or animals exposed to the source, and human cases are among the major control measures for human outbreaks. Treatment of simple cutaneous cases is recommended in an outpatient setting. The control strategies also emphasize animal outbreak control measures, as most human cases are secondary to animal outbreaks. Patients with systemic illnesses should receive antibiotics and supportive treatment^5^.

## Sources of funding

No funding was received.

## Author contribution

K.Z. and A.M.: designed the original draft; R.S.: reviewed the literature and critically edited the manuscript. All authors read and approved the final manuscript.

## Conflicts of interest disclosure

The authors declare no conflicts of interest.

## Provenance and peer review

Not commissioned, externally peer-reviewed.
